# Effects of bone marrow sparing radiotherapy on acute hematologic toxicity for patients with locoregionally advanced cervical cancer: a prospective phase II randomized controlled study

**DOI:** 10.1186/s13014-024-02432-7

**Published:** 2024-04-09

**Authors:** Wen Li, Lan Ma, Fang Li, Kemin Li, Yang Zhang, Hongtao Ren, Xing Bao, Yuyan Guo, Ya Guo, Mincong Wang, Dan Li, Yuanqiong Duan, Xiulong Ma, Zhongwei Wang, Yali Wang, Rutie Yin

**Affiliations:** 1 Department of Obstetrics and Gynecology, West China University Hospital 2, Sichuan University, 610041 Chengdu, China; 2 Key Laboratory of Birth Defects and Related Diseases of Women and Children, Ministry Education, Sichuan University, 610041 Chengdu, China; 3 Department of Radiation Oncology, The Second Affiliated Hospital of Xi ’an Jiaotong University, Xi’ An Jiao Tong University, 710004 Xi’An, China

**Keywords:** Bone marrow sparing (BMS), Hematologic toxicity (HT), Pelvic irradiation, Cervical cancer

## Abstract

**Objective:**

To evaluate effects of bone marrow sparing (BMS) radiotherapy on decreasing the incidence of acute hematologic toxicity (HT) for locoregionally advanced cervical cancer (LACC) patients treated by pelvic irradiation.

**Materials and methods:**

LACC patients were recruited prospectively from May 2021 to May 2022 at a single center and were evenly randomized into the BMS group and the control group. All patients received pelvic irradiation with concurrent cisplatin (40 mg/m2 weekly), followed by brachytherapy and BM V40 < 25% in the BMS group was additionally prescribed. Acute HT was assessed weekly. Binary logistic regression model and receiver operating characteristic (ROC) curve were used for predictive value analysis. The trial was registered with Chinese clinical trial registry (ChiCTR2200066485).

**Results:**

A total of 242 patients were included in the analysis. Baseline demographic, disease and treatment characteristics were balanced between the two groups. In the intention-to-treat population, BMS was associated with a lower incidence of grade ≥ 2 and grade ≥ 3 acute HT, leukopenia and neutropenia s(72.70% v 90.90%, *P* < 0.001*; 16.50% vs. 65.30%, *P* < 0.001*; 66.10% vs. 85.10%, *P* = 0.001*; 13.20% vs. 54.50%, *P* < 0.001*; 37.20% vs. 66.10%, *P* < 0.001*; 10.70% vs. 43.80%, *P* < 0.001*). BMS also resulted in decreased dose delivered to the organs at risk (OARs) including rectum, bladder and left and right femoral head. Univariate and multivariate analyses showed that BM V40 was an independent risk factor for grade ≥ 3 acute HT (odds ratio [OR] = 2.734, 95% confidence interval [CI] = 1.959–3.815, *P* < 0.001*). Cutoff value was 25.036% and area under the curve (AUC) was 0.786. The nomogram was constructed, which was rigorously evaluated and internally cross-validated, showing good predictive performance.

**Conclusions:**

Receiving BMS pelvic irradiation could reduce the incidence of acute HT in LACC patients, and BM V40 < 25% may be a significant factor in reducing the risks of acute HT.

**Supplementary Information:**

The online version contains supplementary material available at 10.1186/s13014-024-02432-7.

## Introduction

The morbidity and mortality rates of cervical cancer remain high among women worldwide, ranking fourth [1]. For locoregionally advanced cervical cancer (LACC) patients, treatment paradigms consist of pelvic irradiation combined with concurrent cisplatin-based chemotherapy, followed by brachytherapy [2, 3]. Nevertheless, concurrent chemoradiotherapy (CCRT) often lead to a high incidence of acute hematological toxicity (HT), which can result in interruptions and prolonged duration of radiotherapy beyond 8 weeks, negatively impacting outcomes of patients [4–6]. Moreover, HT can lead to infection, the need for blood transfusions, bleeding and even immunosuppression, resulting in increased hospitalization rates and treatment costs. The quality of life has been significantly compromised, a factor that holds substantial importance in contemporary cancer treatments [7]. Therefore, it is imperative to identify solutions to reduce the incidence of HT.

Bone marrow (BM) is considered an early response tissue characterized by active division and proliferation, demonstrating a high radiosensitivity similar to that of many tumors, with an alpha/beta ratio of approximately 10. In healthy adults, the active BM predominantly resides within the flat bones—including the skull, sternum, ribs, and iliac bones—as well as the vertebrae, clavicles, scapulae, and the epiphyses of long bones [8]. Patients with LACC undergoing pelvic irradiation will have significant portions of their BM within the radiation field; specifically, regions of the lumbar and sacral spine, hip bone, and both femoral heads are exposed, encompassing an estimated 51% of the active BM [9]. Dose restrictions are applied to the femoral heads, which account for around 4% of the active BM, to prevent complications such as necrosis and fractures. Notably, the pelvic BM is not typically included among the organs at risk (OARs) that are routinely protected during radiotherapy.

Imaging studies have revealed that BM damage is pronounced following pelvic irradiation [10]. Exposure to ionizing radiation (IR) leads to double-strand DNA breaks (DSBs), resulting in cell death. Direct damage arising from immediate interaction with irradiation and indirect damages caused by reactive oxygen species (ROS) are two primary mechanisms of IR-induced DNA damage [11]. Researches have demonstrated that the production of ROS within irradiated BM can be augmented by various sources, including nicotinamide adenine dinucleotide phosphate oxidases (NOXs), cyclooxygenases, mitochondrial electron transport chain-1 [12–15]. Nitric oxide (NO) is another mediator for division, survival and mobilization of hematopoietic stem and progenitor cells and the production of NO is known to increase in irradiated BM, further contributing to BM damages [16].

Injury to localized hematopoietic cells resulting from pelvic irradiation can be efficiently repaired through the recruitment of circulating pluripotent hematopoietic stem cells [17]. But concurrent chemotherapy can synergistically exacerbate such injuries, leading to acute HT. BM is a parallel organ and it is effective to limit local radiation dose, consequently pelvic BM sparing (BMS) is a promising strategy to mitigate acute HT. By limiting the radiation dose to the pelvic BM, both pathologic and radiographic damage can be lessened, and the incidence of acute HT can be significantly decreased [18, 19].

BMS has been implemented not only in cervical cancer, but also other pelvic malignancies, such as rectum cancer and anal cancer, which turned out to be effective in decreasing the incidence of acute HT [20–23]. Nevertheless, the importance of BM as an OAR has received little attention in the radiotherapy protocols for prostate and endometrial cancer [24].

The target volume of radiotherapy, despite optimization efforts, inevitably involves normal tissue to some extent. Advancements in radiation technologies serve as an effective strategy to alleviate toxicity. Intensity modulated radiotherapy (IMRT) allows beams to be segmented into thousands of tiny pencil-thin radiation beams, each with varying intensities entering the body from multiple angles, thus enabling the irradiation of complex, irregular clinical target volumes with sparing of the adjacent normal tissues. Compared to the now infrequently used conventional two-dimensional four-field pelvic radiotherapy and three-dimensional conformal radiotherapy (3DCRT), IMRT has been shown to reduce the incidence of HT [25, 26].

In two-dimensional four-field pelvic radiotherapy, 3DCRT and IMRT, BM within the treatment plan have been delineated based on the bone’s outer contour, including both active and inactive BM [25–27]. Image-guided IMRT (IG-IMRT) integrates with single photon emission computed tomography (SPECT)/computed tomography (CT) or 18 F-fluorodeoxyglucose positron emission tomography (18FDG-PET)/CT, which allows for PET-based BMS [28–30]. This strategy spares myeloid precursors concentrated in active BM, guided by the standard uptake value (SUV) [31]. Nevertheless, the utilization of PET in newly diagnosed cervical cancer patients with biopsy-proven carcinoma of the cervix is still limited. Given the widespread application of IMRT, here we conducted a prospective randomized controlled trial (RCT) to evaluate the effects of BMS-IMRT on acute HT in LACC patients.

## Materials and methods

### Patients

From May 2021 to May 2022, eligible LACC patients were recruited for the RCT study, who received pelvic IMRT and cisplatin-based concurrent chemotherapy, followed by brachytherapy.

The Institutional Review Board of the Second Affiliated Hospital of Xi’an Jiao Tong University approved the clinical trial (approval number: 2021-021), and the trial was conducted in accordance with the Declaration of Helsinki and Good Clinical Practice guidelines [32]. Prior to the intervention, all patients were thoroughly informed about the study design and potential risks, and provided signed informed consent. The trial was registered on Chinese Clinical Trial Registry with the identifier ChiCTR2200066485.

Eligibility criteria included: (1) Patients aged between 18 and 75 years with Karnofsky performance status (KPS) ≥ 70. (2) Cervical biopsy-proven squamous cell carcinoma, adenocarcinoma, or adenosquamous carcinoma of the cervix. (3) International Federation of Gynecology and Obstetrics (FIGO, 2018) stage IB3, IIA2-IVA. (4) Absence of serious underlying medical conditions, including hematopoietic abnormalities and abnormal heart, lung, liver, kidney function, or immunodeficiency.

Exclusion criteria included: (1) Patients with a history of hematologic diseases or malignancies other than cervical cancer. (2) Patients who have previously received surgical treatment for cervical cancer, pelvic irradiation, interventional therapy, chemotherapy, targeted therapy, or immunotherapy. (3) Patients who were pregnant or breastfeeding. (4)Patients presenting with uncontrolled vaginal bleeding, a risk of vaginal fistula formation, or ureteral obstruction. (5) Patients concurrently enrolled in other clinical trials.

### External beam radiotherapy (EBRT)

#### CT-based simulation

A Pelvic fixation plate and a thermoplastic mold were utilized to ensure repeatability of the EBRT procedure, and patients were immobilized in the horizontal supine position with their arms raised above their heads. A contrast-enhanced CT scan with a slice thickness of 5 mm was performed from the T12-L1 interspace to 5 cm below ischial tuberosity.

#### Definition of target volume and OARs

The CT image datasets were imported into the treatment planning system (TPS). The target volume and OARs were delineated by a consistent deputy chief physician following the International commission on radiation units and measurements (ICRU) related reports [33]. The gross tumor volume node (GTVnd) was defined as a lymph node with short-axis diameter ≥ 1 cm on imaging. The clinical target volume (CTV) included the gross tumor, the cervix, the entire uterus, the parauterine, the vagina, and pelvic lymph node drainage areas. The pelvic lymph node drainage areas included a 7 mm region extending beyond the edge of the blood vessel, encompassing the common iliac, extra ilia, intra ilia as well as the obturator and the presacral regions. The upper boundary of CTV was the bifurcation of the common iliac artery and extending to the renal vascular level when the para-aortic lymph nodes were incorporated into GTVnd. The lower boundary of CTV was set 3 cm beneath the most inferior vaginal involvement. The planning target volume (PTV) of the CTV was expanded by 7 mm laterally and 15 mm axially from the primary CTV; the PTV of the GTVnd (PGTVnd) was enlarged by 7 mm laterally and 15 mm axially on primary GTVnd. OARs included rectum, bladder, femoral head, spinal cord, small intestine and colon. When the upper limit of CTV extended to the renal vascular level, kidney and liver were also considered as OARs.

#### BM contouring

In the BMS group, for each patient, the outer contour of the pelvic bone, lumbar spine, and femoral heads were delineated as a surrogate for BM, based on the CT simulation positioning images in the TPS. The delineation extended from 4 levels above PTV to the end of the ischial nodule.

**Fig. 1:**
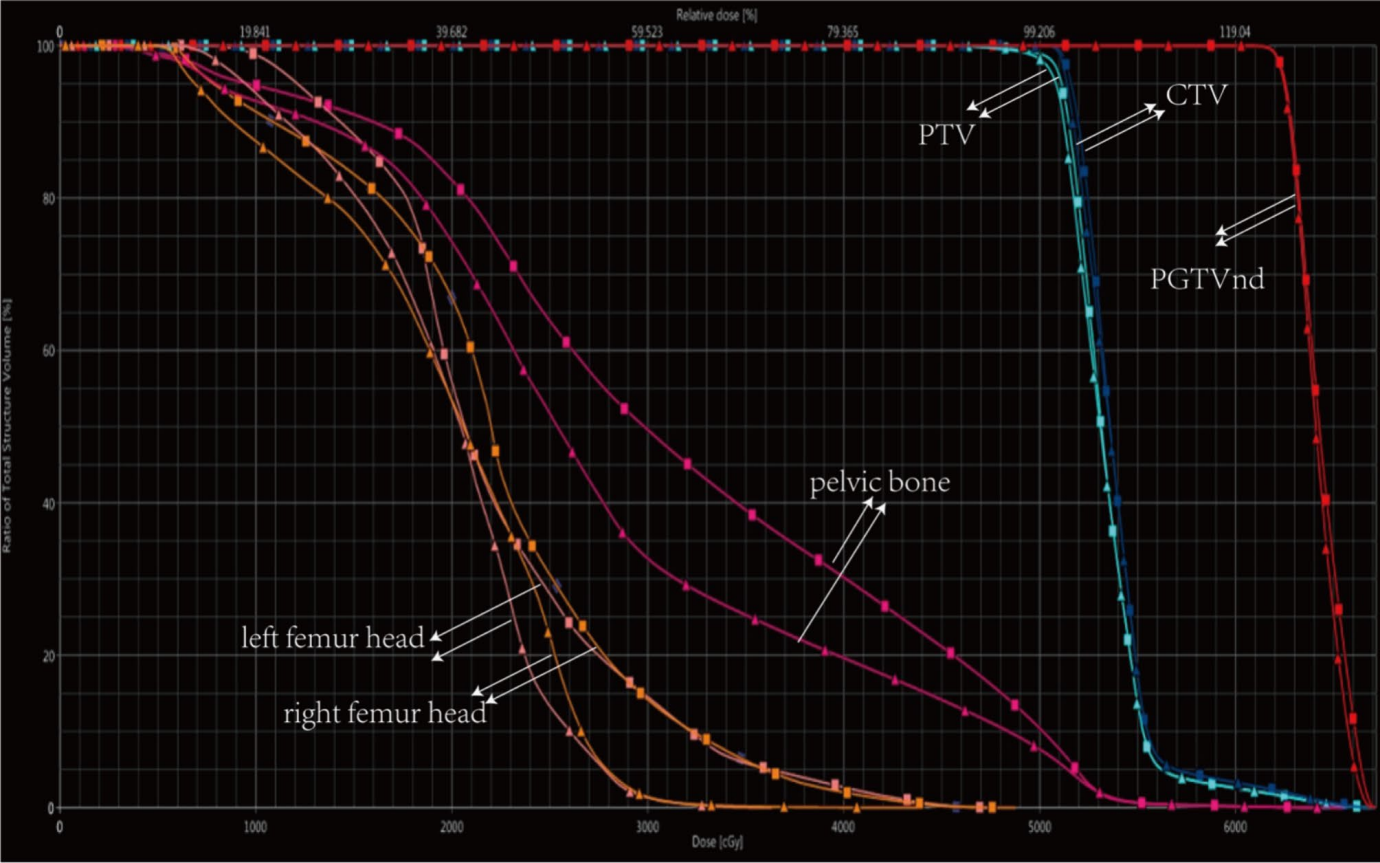
A dual-plan DVH plot of an example patient. The small triangle solid line represented the BMS group, and the small square solid line represented the control group. *Abbreviation* CTV, clinical target volume; DVH, the dose volume histograms; PTV, plan target volume; GTVnd, plan gross tumor volume node

Previously, dual EBRT plans were designed for patients, using pelvic dose gradients, specifically V10, V20, V25, V30 and V40. It was observed that limiting the dose to V40 < 25% resulted in plan conformity and uniformity comparable to that of non-BMS plans, while still meeting the requirements for PTV and OARs (Fig. 1). Consequently, the BM V40 < 25% was prescribed for BMS group. In addition, it was considered that dose to the pelvic BM from intracavitary brachytherapy (ICBT) was negligible.

#### EBRT planning and evaluation

IMRT treatment plans for all patients were developed using TPS. Treatment was administered using Varian 21EX or Trilogy linear accelerators with 6 MV X-rays. Fixed-field static reverse IMRT was implemented with a field number of 5 or 7 coplanar fields. The prescribed dose ranged from 45.0 to 50.4 Gy delivered in 25–28 daily fractions of 1.8-2 Gy per day, five days per week, and PTV was optimized to ≥ 95% of the V(100%). For patients with positive lymph nodes, simultaneous integrated boost (SIB) was used, increasing the dose for PGTVnd to 55-61.6 Gy delivered in 25–28 daily fractions of 2.2 Gy per day. For OARs, it was prescribed that small intestine and colon V40 ≤ 30%, V50 ≤ 10%, rectal and bladder V50 ≤ 50%, left and right femoral heads V50 ≤ 5% respectively, left and right kidneys Dmean ≤ 13 Gy respectively, liver D30% ≤20 Gy, spinal cord V40 ≤ 0%.

The EBRT plan for each patient was designed by a consistent medical physicist, and was evaluated using data from dose volume histograms (DVHs). We also assessed the plan’s conformity and homogeneity. We calculated conformity index (CI) and homogeneity index (HI) as follows: CI = (VPTV, ref/VPTV)× (VPTV, ref /Vref), where VPTV, ref represents the volume of reference isodose surface encompassing the target, VPTV represents the target volume, and Vref represents the volume of reference isodose surface. An optimal conformity is indicated by a CI value closer to 1. HI = [D2% - D99% ]/D50%, where D2%, D50% and D99% correspond to the doses received by 2%, 50% and 99% of the target volume of the. Optimal homogeneity is reflected by a lower HI value.

### ICBT

ICBT was administered to patients using either a two-dimensional therapy technique or image-guided brachytherapy (IGBT) technology. ICBT initiated after the delivery 40–50 Gy of EBRT. A high-dose-rate (HDR) 192iridium after-loading treatment machine was utilized with a dose fractionation schedule of either 6 Gy × 5 fractions or 7 Gy × 4 fractions, 1 to 2 times per week. EBRT and ICBT were typically completed within a duration of 6–8 weeks. In the two-dimensional therapy, point A was defined as a location 2 cm above the ectocervix and 2 cm lateral to the midline. Rectal and bladder were prescribed as Dmax ≤ 60-70%. In the IGBT, High risk CTV (HR-CTV) included the cervix, the vagina and any remaining tumor. It was prescribed that HR-CTV V(100%) ≥ 90%, rectum and bladder D_2cm_^3^ ≤ 75%. The equivalent dose in 2 Gy fractions (EQD2) for point A and HR-CTV was 80–85 Gy, increasing to 87 Gy or higher when the tumor diameter of the is 4 cm or greater.

### Chemotherapy regime

Concurrently with radiotherapy, patients were administered weekly cisplatin-based chemotherapy (40 mg/m2) for a duration of 6 weeks. Patients with FIGO stage III and IVA received 2 to 4 cycles of adjuvant chemotherapy following the completion of radiotherapy. Adjuvant chemotherapy consisted of TP regimen (cisplatin 60 mg/m2 d1-2 + paclitaxel 135 mg/m2 d1), once every 21 days. Chemotherapy and radiotherapy were typically held if the white blood cell count (WBC) was < 2.0 × 109/L, the absolute neutrophil cell count (ANC) was < 1.0 × 109/L, or the platelet (PLT) count was < 50 × 109/L. Chemotherapy was additionally held if patients developed febrile neutropenia, renal failure, grade ≥ 2 neurotoxicity, or grade ≥ 3 nausea lasting > 24 h. Acute toxicity was graded using the National Cancer Institute Common Terminology Criteria for Adverse Events (CTCTE, version 4.03) [34].

### Study endpoint

The primary endpoint of this phase II randomized trial was HT during EBRT. Blood tests including WBC, ANC, hemoglobin (HGB), PLT, and lymphocyte (LYM), were conducted at least weekly throughout the treatment course, with the nadir observed during EBRT used to assess HT. Acute HT was graded according to CTCAE (version 4.03, Table 1s) [34].

Secondary endpoint included dosimetric parameters from EBRT plan’s DVHs, including PTV V(100%), CI, HI, small intestine V40 (%) and V50 (%), rectum V50(%), bladder V50(%), left and right femoral head V30 (%) and V50 (%), left and righ kidney Dmean (Gy), liver D(30%), spinal cord V40(%), and BM V10(%) to V50(%).

### Study design

This study was conducted as an open-label, single-center, prospective, randomized clinical trial. Participant enrollment was achieved within a timeframe of 12 months, followed by a 3-month follow-up period. Prior to the initiation of treatment, eligible participants were randomized into two groups (BMS and control), utilizing a computer-generated random number list. Based on previously published studies, we estimated an incidence of grade ≥ 3 acute HT in the BMS group of 40%. The incidence rate of grade ≥ 3 acute HT in the control group was estimated at 62% according to a pretest in our center. To achieve a power of 90% and a two-sided type I error of 0.05, the required sample size for each group was 104 according, based on a two-sided test. Considering an anticipated maximum loss to follow-up rate of approximately 20% for this trial, the final sample size was adjusted to 127 participants for each group.

### Statistical analysis

SPSS (IBM, Chicago, IL, version 28.0), R (version 4.1.0, http://www.rproject.org/) and GraphPad Prism (version 9.0) software were used for statistical analysis and to generate graphs for the study. Continuous variables were compared with parametric methods when a normal distribution was confirmed. For non-normally distributed variables and categorical data, nonparametric tests were utilized for comparisons. Univariate and multivariate analyses were conducted using a binary logistic regression model. The receiver operating characteristic (ROC) curve was used for predictive value analysis. The “rms” package in R was used to generate the nomograms. Unless otherwise stated, all analyses were performed with a 2-sided significance level of *P* = 0.05.

## Results

### Patient characteristics

**Fig. 2:**
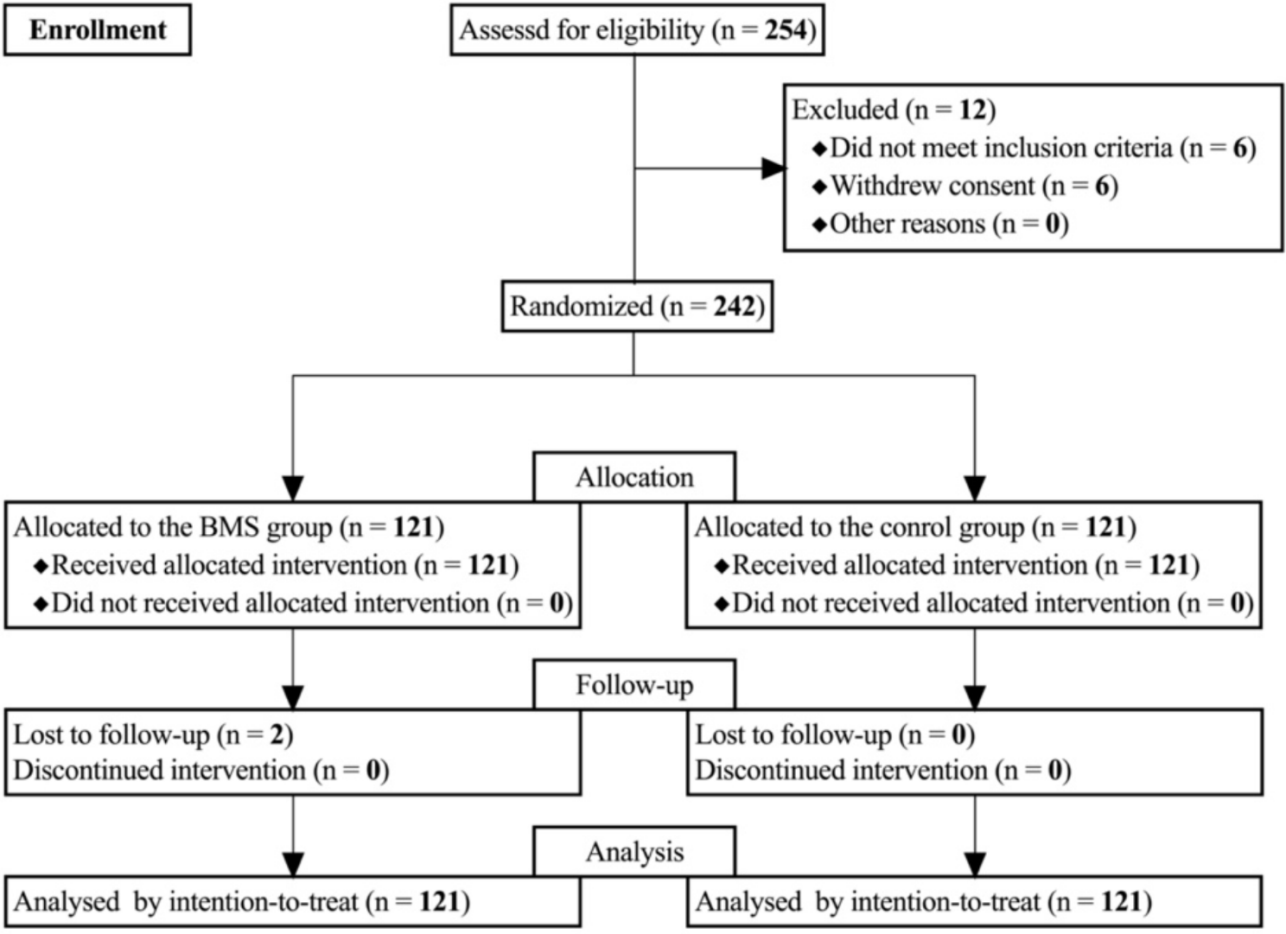
Randomization and intervention of the cohort

A total of 254 eligible LACC patients were enrolled in this study (Fig. 2). Of these, 6 patients withdrew their consent and another 6 did not meet the inclusion criteria (3 patients for hemoglobin < 10 g/dL; 2 patients for cervical lymphatic metastasis diagnosed by aspiration biopsy). Consequently, based on a computer-generated random number list, 242 patients were assigned to the BMS group (*n* = 121) and the control group (*n* = 121), all of whom were subsequently included in the intention-to-treat analysis. 2 patients in the BMS group were lost to follow-up.

Baseline demographic parameters and chemotherapy cycles received were balanced between two treatment groups (Table 1). All patients received a minimum of four cycles of concurrent chemotherapy. In the BMS group, 42 (34.70%) patients received a 45 Gy/25f dose of EBRT, 53 (43.80%) patients received 50 Gy/25f, and 26 (21.50%) received 50.4 Gy/28 f. In the control group, it was 45 (37.20%), 51 (42.10%) and 25 (20.70%) patients respectively. No significant difference was observed in the EBRT dose distribution between two groups. Similarly, no significant difference was observed in other radiotherapy variables, including the prescribed doses of SIB and ICBT. Furthermore, baseline routine blood test results, including WBC, ANC, HGB, PLT and LYM and HT (excluding lymphopenia) demonstrated no significant difference between the two groups.

**Table 1:** Clinicopathological and treatment characteristics of patients

Characteristics	BMS group	Control group	P
	n(%)	n(%)	
No	121	121	
Age,(mean, s.d)	57.21(10.63)	55.73(10.45)	0.276
Range	28–75	20–72	
< 60	72(59.50%)	81(66.90%)	0.230
≥ 60	49(40.50%)	40(33.10%)	
KPS			
70	0	0	0.511
80	3(2.50%)	5(4.10%)	
90	48(39.70%)	54(44.60%)	
100	70(57.90%)	62(51.20%)	
FIGO stage			
IB3-II	74(61.20%)	68(56.20%)	0.433
III-IVA	47(38.80%)	53(43.80%)	
in detail			
IB3	38(31.40%)	26(21.50%)	0.210
IIA2	15(12.40%)	10(8.30%)	
IIB	21(17.40%)	32(26.40%)	
IIIA	1(0.80%)	3(2.50%)	
IIIB	3(2.50%)	4(3.30%)	
IIIC1	26(21.50%)	29(24.00%)	
IIIC2	11(9.10%)	15(12.40%)	
IVA	6(5.00%)	2(1.70%)	
Histologic type			
Squamous cell carcinoma	103(85.10%)	105(86.80%)	0.870
Adenocarcinoma	13(10.70%)	13(10.70%)	
Adenosquamous carcinoma	5(4.10%)	3(2.50%)	
Concurrent chemotherapy cycles received			
4	9(7.40%)	7(5.80%)	0.719
5	33(27.30%)	38(31.40%)	
6	79(65.30%)	76(62.80%)	
Adjuvant chemotherapy cycles received			
0	69(57.00%)	63(52.10%)	0.719
2	5(4.10%)	8(6.60%)	
3	1(0.80%)	2(1.70%)	
4	46(38.00%)	48(39.70%)	
EBRT dose			
45 Gy/25f	42(34.70%)	45(37.20%)	0.922
50 Gy/25f	53(43.80%)	51(42.10%)	
50.4 Gy/28f	26(21.50%)	25(20.70%)	
SIB prescribed			
50 Gy/25f	14(11.60%)	21(17.40%)	0.441
61.6 Gy/28f	26(21.50%)	24(19.80%)	
No	81(66.90%)	76(62.80%)	
ICBT dose			
30 Gy/5f	76(62.80%)	72(59.50%)	0.598
28 Gy/4f	45(37.20%)	49(40.50%)	
Baseline blood count test (mean, s.d)			
WBC, *10^9/L	6.39(3.74)	6.32(3.76)	0.887
ANC, *10^9/L	4.60(3.26)	4.43(3.51)	0.704
HGB, g/L	120.60(15.08)	118.10(14.80)	0.194
PLT, *10^9/L	214.03(78.92)	208.98(82.23)	0.626
LYM,*10^9/L	1.37(0.59)	1.41(0.61)	0.663
Baseline HT(excluding lymphopenia)			
Grade 0	53(43.80%)	47(38.8%)	0.112
Grade 1	61(50.4%)	54(44.6%)	
Grade 2	7(5.8%)	20(16.5%)	
Grade 3	0	0	
Grade 4	0	0	

*Abbreviation* BMS, bone marrow-sparing; s.d. standard deviation; KPS, Karnofsky performance status; FIGO, International Federation of Gynecology and Obstetrics; EBRT, External beam radiotherapy; SIB, simultaneous integrated boost; ICBT, Intracavitary brachytherapy; WBC, White blood cell; ANC, absolute neutrophil cell; HGB, hemoglobin; PLT, platelet; LYM, lymphocyte. * *P* < 0.05 was considered significant

### Effects of BMS on acute HT

For patients enrolled in the trial, blood count tests were performed weekly, and nadirs during EBRT were used as data. In total, 8 (3.30%) patients did not experience any acute HT (excluding lymphopenia). 36 (14.90%), 99 (40.90%), 69 (28.50%) and 30 (12.40%) patients experienced grade 1, grade 2, grade 3 and grade 4 HT separately (Fig. 1s). Treatment was interrupted for HT in 94 patients, after which chemotherapy and radiotherapy resumed once the toxicity subsided.

Patients in the BMS group were primarily in grades 0–2 HT, while those in the control group were mostly in the grade 2–4. The incidence of grade ≥ 2 and grade ≥ 3 acute HT, leukopenia and neutropenia was significantly lower in the BMS group as compared with the control group (72.70% vs. 90.90%, *P* < 0.001*; 16.50% vs. 65.30%, *P* < 0.001*; 66.10% vs. 85.10%, *P* = 0.001*; 13.20% vs. 54.50%, *P* < 0.001*; 37.20% vs. 66.10%, *P* < 0.001*; 10.70% vs. 43.80%, *P* < 0.001*) (Table 2). In cases of anemia and thrombocytopenia, the incidence of grade ≥ 2 toxicity was significantly reduced in the BMS group (17.40% vs. 30.60%, *P* = 0.016*; 20.70% vs. 33.10%, *P* = 0.030*). For grade ≥ 2 and grade ≥ 3 lymphopenia, no significant difference was observed. When we included incidence of lymphopenia in acute HT, difference showed no significance for grade ≥ 2 and grade ≥ 3 toxicity between the two groups.

**Fig. 3:**
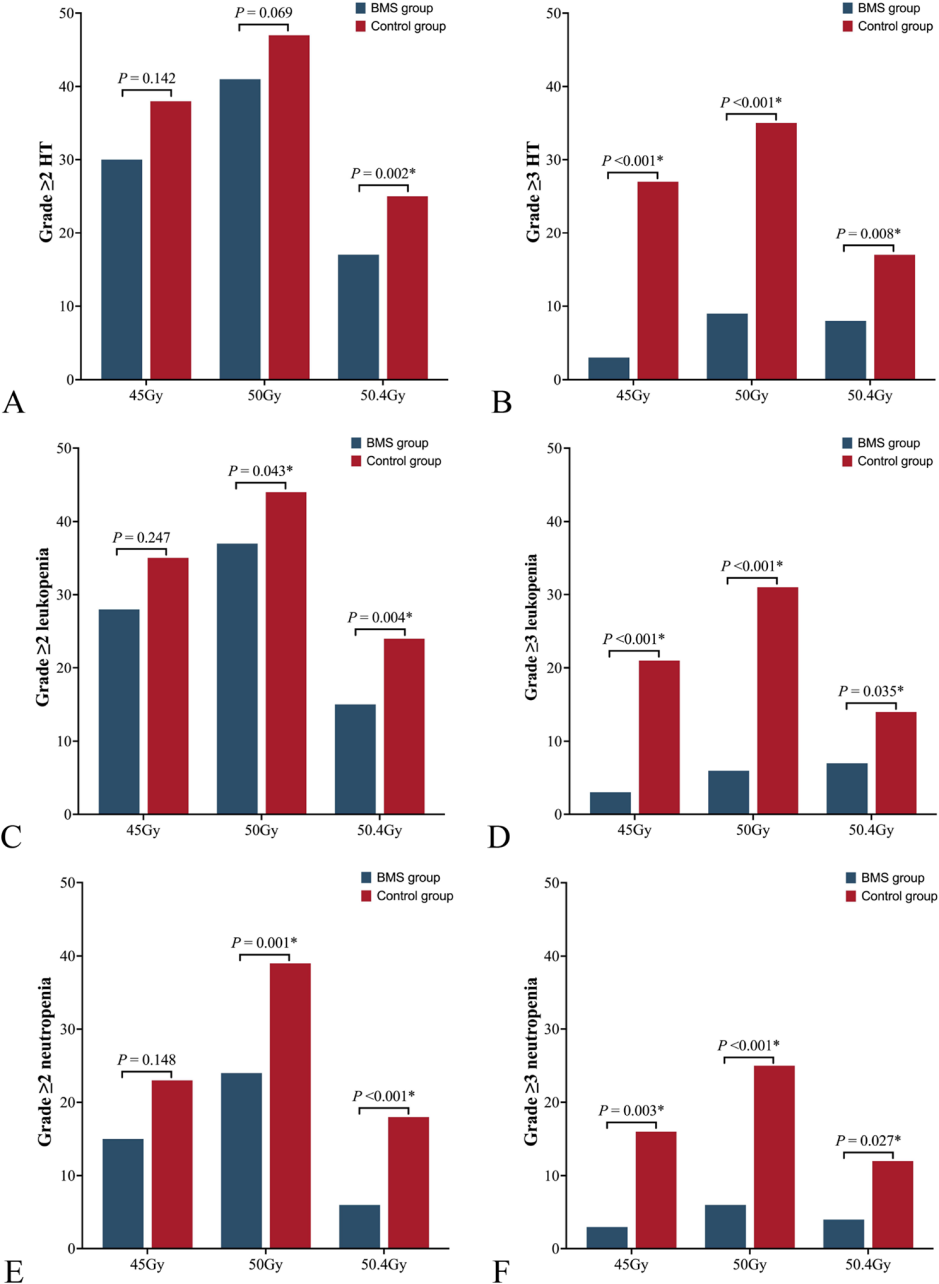
Comparison of incidence of grade ≥ 2 and grade ≥ 3 toxicity between the BMS group and control group. (**A-B**) Acute HT excluding lymphopenia. (**C-D**) Leukopenia. (**E-F**) Neutropenia. *Abbreviation* HT, hematologic toxicity; BMS, bone marrow sparing; ns, no significance. * *P* < 0.05 was considered significant

Patients were stratified into three groups based on the administered EBRT dose,and the incidence of HT across the groups was compared. The distribution of patients was 42 vs. 45, 53 vs. 51, and 26 vs. 25 for the BMS and control groups, respectively, corresponding to EBRT doses of 45 Gy/25f, 25 Gy/25f, and 50.4 Gy/28 f. Incidences of grade ≥ 2 or grade ≥ 3 acute HT (excluding lymphopenia), leukopenia and neutropenia were significantly reduced in the BMS group compared to the control group (Tables 2, 3 and 4 s, Fig. 3A-F). Incidences of Grade ≥ 2 and grade ≥ 3 anemia, thrombocytopenia, lymphopenia and acute HT (including lymphopenia) were comparable between the groups.

**Table 2:** Grade ≥ 2 and grade ≥ 3 acute HT(excluding and including lymphopenia), leukopenia, neutropenia, anemia, thrombocytopenia and lymphopenia for all patients

	Grade ≥ 2			Grade ≥ 3		
	BMS (*n* = 121)	Control (*n* = 121)	P	BMS (*n* = 121)	Control (*n* = 121)	P
HT(excluding lymphopenia)	88(72.70%)	110(90.90%)	*P* < 0.001*	20(16.50%)	79(65.30%)	*P* < 0.001*
Leukopenia	80(66.10%)	103(85.10%)	0.001*	16(13.20%)	66(54.50%)	*P* < 0.001*
Neutropenia	45(37.20%)	80(66.10%)	*P* < 0.001*	13(10.70%)	53(43.80%)	*P* < 0.001*
Anemia	21(17.40%)	37(30.60%)	0.016*	5(4.10%)	11(9.10%)	0.121
Thrombocytopenia	25(20.70%)	40(33.10%)	0.030*	5(4.10%)	8(6.6%)	0.392
Lymphopenia	119(98.30%)	120(99.20%)	1.000	109(90.10%)	107(88.40%)	0.678
HT(including lymphopenia)	121(100.00%)	120(99.20%)	1.000	110(90.90%)	116(95.90%)	0.121

*Abbreviation* HT, hematologic toxicity; BMS, bone marrow sparing; ns, no significance. * *P* < 0.05 was considered significant

### Evaluation of PTV and OARs

Dosimetric parameters for PTV and OARs were compared between the two groups (Table 5s). PTV V(100%) was significantly lower in the BMS group than in the control group (96.27% ± 1.07% vs. 96.75% ± 1.29%, *P* = 0.002*). However, the dose distribution within the PTV for each patient met the clinical requirement of at least 95% of the PTV volume receiving 100% or more of the prescribed dose. CI and HI were both comparable between the two groups. For OARs, the results were encouraging as parameters of rectum, bladder and left and right femoral heads showed a significant decrease in the BMS group compared to the control group. Dosimetric parameters for the small intestine, both kidneys, and liver demonstrated no increase in the BMS group. Spinal cord V40(%) in the two groups both satisfied requirements.

Dosimetric parameters of BM ranging from to V10(%) to V50(%) were displayed and their median values were presented (Fig. 2s-A). Median value of BM V40(%) was 24.95%. Additionally, we compared BM V10(%) to V50(%) between the BMS group and the control group. With the exception of V10(%) and V15(%), all other parameters for BMS group were higher than those for the control group(Fig. 2s-B).

### Analysis of factors for acute HT

**Fig. 4:**
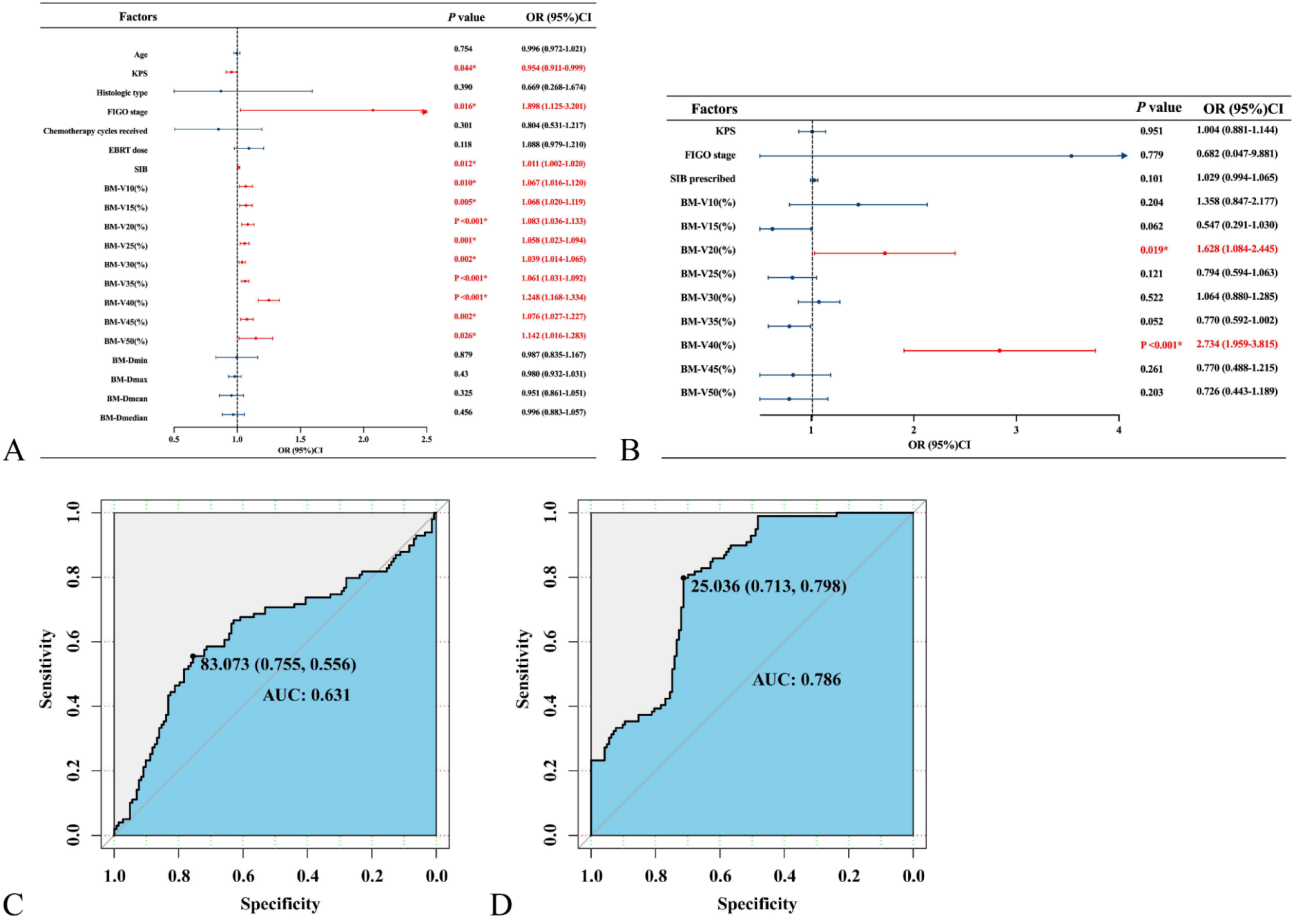
Independent risk factors analyses for grade ≥ 3 acute HT. Univariate (**A**) and multivariate (**B**) binary logistic regression analyses. Receiver operating characteristic (ROC) curves for BM V20 (**C**) and V40 (**D**). *Abbreviation* OR, odds ratio; CI, confidence interval; KPS, Karnofsky performance status; FIGO, International Federation of Gynecology and Obstetrics; EBRT, External beam radiotherapy; SIB, simultaneous integrated boost; BM, bone marrow; Vx,volume receiving x Gy; Dmin, minimum dose; Dmax, maximize dose, Dmean, mean dose; Dmedian, median dose; AUC, under the curve. * *P* < 0.05 was considered significant

To identify clinical and dosimestric factors for grade ≥ 3 acute HT, we conducted stepwise univariate and multivariate binary logistic regression analyses (Fig. 4A-B). Clinical factors encompassed age, KPS, histologic type and chemotherapy cycles received. Dosimestric factors comprised EBRT dose, SIB prescribed, V10-50 as well as minimum dose (Dmin), maximize dose (Dmax), Dmean and Dmedian of BM. Univariate analysis showed that KPS, FIGO stage, SIB prescribed and all dosimetric parameters of BM were associated with grade ≥ 3 HT. Multivariate analysis showed that BM V20 and V40 were predictors of risk (odds ratio [OR] = 1.628, 95% confidence interval [CI] = 1.084–2.445, *P* = 0.019*; OR = 2.734, 95%CI = 1.959–3.815, *P* < 0.001*). Each incremental unit of BM V20 or V30 was associated with a 62.8% or 173.4% increased risk of grade ≥ 3 acute HT (excluding lymphopenia). We generated the ROC curves for grade ≥ 3 HT and BM V20, V30 (Fig. 4C-D). For V20, the cutoff value was 83.073% with an area under the curve (AUC) of 0.631. The sensitivity and specificity were 0.755 and 0.556, respectively. For V40, the cutoff value was 25.036% with an AUC of 0.786. The sensitivity and specificity were 0.713 and 0.798, respectively.

**Fig. 5:**
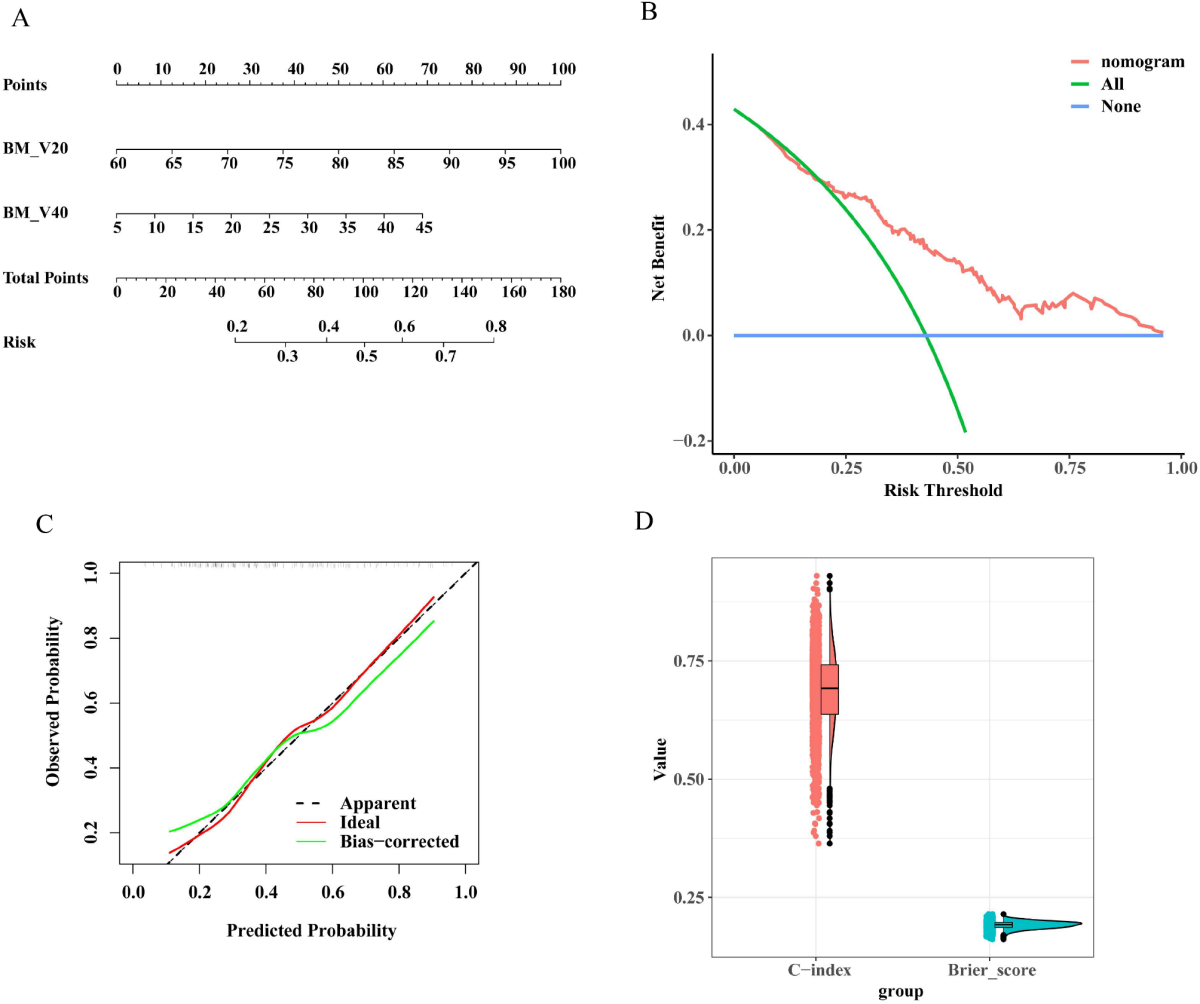
Construction and evaluation of the nomogram. (**A**) Nomogram. (**B**) Calibration curve. (**C**) Decision Curve Analysis (DCA) curve. (**D**) 2000 times five-fold cross-validated C-indexes and Brier scores. *Abbreviation* BM, bone marrow; Vx,volume receiving x Gy; C-index, concordance index. * *P* < 0.05 was considered significant

### Construction and evaluation of the nomogram

We developed a prognostic nomogram based on the independent factors identified through logistic regression analyses (Fig. 5A). The nomogram quantifed each predictor, roviding an individualized calculation of a patient’s total risk score, corresponding to the probability of grade ≥ 3 acute HT (excluding lymphopenia). The concordance index (C-index) was 0.759, and the Brier score was 0.195 A calibration curve was plotted to evaluate the consistency between the predicted and actual risk, which closely followed the 45° line, indicating good consistency (Fig. 5B). Decision Curve Analysis (DCA) demonstrated the nomogram’s good clinical utility(Fig. 5C). When the threshold probability for grade ≥ 3 HT ranged from 0.033 to 0.960, the net benefit of applying the nomogram (ranging from 0.505 to 40.983%) was significantly greater than that of the “no intervention” and “intervention for all” strategies. Furthermore, we performed 2000 times five-fold cross-validation for the C-index and Brier score, yielding a median C-index of 0.692 (interquartile range [IQR] = 0.637–0.742) and Brier score of 0.192 (IQR = 0.187–0.197) (Fig. 5D).

## Discussion

It is generally accepted that EBRT and ICBT should be completed within 8 weeks, as each additional day of RT duration results in a 0.5–0.1% reduction in pelvic control [5, 35]. However, acute HT can interrupt treatment and prolong the duration, a frequent adverse effect in LACC patients undergoing CCRT. Compared with pelvic irradiation alone, CCRT remarkably improves overall survival and concurrently reduces the rates of recurrence and metastasis [4]. At the same time, the incidence of acute HT also rises, as irradiation damages not only hematopoietic cells in the pelvic BM but also those throughout the entire body due to systematic chemotherapy [36]. Encopassing a substantial patient cohort, our study verified that BMS can reduce the incidence of acute HT, which is paticularly vital for LACC patients receiving CCRT to complete the therapeutic plan timely.

Multiple studies have reported that BMS can reduce the incidence of grade ≥ 2 or grade ≥ 3 HT. However, the benefits of BMS to date have been complicated by the use of varied EBRT techniques, inconsistent definition of BM, heterogeneous trial results, and diverse control group configuration. Studies by Hui et al. and Chang et al. proved that IMRT results in milder HT than 3DCRT [26, 37]. In IMRT era, Radiation Therapy Oncology Group (RTOG) 0418 research concluded that BM V40 > 37% or Dmedian > 34.2 was correlated with a higher rate of grade ≥ 2 HT [18]. Huang et al. recommended that efforts to maintain lumbosacral spine (LSS) V10 < 87%, LSS Dmean < 39 Gy and pelvic bone (PB) V40 < 28% simultaneously may reduce the risk of grade ≥ 2 HT [38]. It was reported also that patients with BM V10 ≥ 95% or V20 > 76% were more likely to experience grade ≥ 3 leukopenia [19]. In patients with rectum cancer, Newman et al. found that pelvic mean dose ≥ 36.6 Gy in neoadjuvant IMRT was strongly correlated with the occurrence of grade3 HT during postoperative chemotherapy [21]. In patients with anal cancer, Lee et al. reported that patients receiving CCRT with BM V40 > 23% experienced a higher rate of grade ≥ 3 leukopenia [22]. However, for endometrial patients receiving postoperative IMRT alone, BM volume was not correlated significantly with the incidence of acute HT [18]. As radiation technology improved, more studies were performed to explore the effect of PET-based BMS on reducing the incidence of HT. Mell et al. defined active BM as the subregion with a SUV on PET/CT greater than the mean value over the total BM volume [39]. When pelvic marrow and active marrow mean dose were limited to < 27 Gy and < 28.5 Gy, respectively, along with V10 < 90% and V20 < 75%, the research proved that PET-BMS-IMRT significantly reduced the incidence of grade ≥ 3 neutropenia compared with standard IMRT. Khullar et al. also concluded that a lower volume of active BM defined by PET at a baseline (< 1201 mL) was highly predictive of grade ≥ 3 HT [40].

Our findings add to a large body of evidence that the incidence of acute HT can be decreased in LACC patients receiving BMS pelvic irradiation. Patients in our study were treated with IMRT and we delineated the entire pelvic bone as BM. Compared with previous studies, we included the incidence of leukopenia, neutropenia, anemia, thrombocytopenia, as well as acute HT involving the four variables above as observed results. Additionally, lymphopenia and HT including lymphopenia were analyzed. Our results indicated that the incidence of grade ≥ 2 and grade ≥ 3 acute HT excluding lymphopenia, leukopenia and neutropenia was significantly lower in the BMS group, aligning with the previously reported results. Furthermore, when patients were stratified into 3 groups according to the EBRT dose, a reduction in grade ≥ 2 or grade ≥ 3 HT was also observed in the BMS group. But the incidence of HT including lymphopenia and lymphopenia was comparable between the two groups. PTV V(100%) of all patients met the goal of ≥ 95% and no significance was observed in conformity and homogeneity between the two groups. For OARs, dosimetric parameters of patients in the BMS group were comparable or superior. It was concluded that BMS-IMRT was highly feasible. Notably, rectum V50, bladder V50, left and right femoral head V30 decreased in the BMS group compared with the control group, potentially reducing the incidence of radiation-induced proctitis, cystitis, femoral head and neck injuries. Previous studies had scarcely reported a decrease in the dose to OARs. Subsequent logistic regression analyses proved that BM V20 and V40 were independent risk factors. ROC analysis further corroborated our findings that BM V40 < 25.036% was significantly correlated with a lower incidence of grade ≥ 3 acute HT, with an AUC of 0.786. Ultimately, a nomogram was constructed and rigorously evaluated through internal cross-validation, exhibiting robust predictive performance.

Our results corroborate the reported results with significantly enhanced practical and clinical applicability regarding the EBRT techniques and BM definition. Financial predicament and technological challenges, especially in low- and middle-income countries, hinder the widespread adoption of IG-IMRT. BMS-IMRT is a promising strategy to improve acute HT, as it is both effective and cost-effective.

Our study had several limitations. First, the single-centered study may introduce regional bias. Second, although the dose to rectum, bladder, and femoral head were reduced, we did not include the incidence of complications of pelvic irradiation. Third, survival of patients and pelvic insufficiency fracture (PIF) caused by pelvic irradiation were not assessed due to the short duration of follow-up. We are currently following up patients to observe whether BMS-IMRT confers survival benefits or decrease the incidence of PIF. Fourth, our study was limited to patients with cervical cancer and did not encompass individuals with other pelvic malignancies receiving pelvic irradiation.

## Conclusion

In patients with LACC receiving CCRT, BMS exhibited significant benefits in the incidence of acute HT, without compromising the efficacy of radiotherapy or damaging adjacent normal tissues. BMS takes on significance to balance the benefits and risks. Multicenter study, long-term follow-up and the inclusion of other pelvic malignancies are required in future investigations.

### Electronic supplementary material

Below is the link to the electronic supplementary material.

Supplementary Material 1

Supplementary Material 2

## Data Availability

Research data are stored in an institutional repository and will be shared upon request to the corresponding author.

## Declarations

### Clinical trial information

The study was approved by the Second Affiliated Hospital of Xi’an Jiao Tong University Institutional Review Board (2021-021) and was conducted in compliance with the Declaration of Helsinki and Good Clinical Practice guidelines. All patients provided informed consent. And the study was registered on Chinese Clinical Trial Registry with the identifier ChiCTR2200066485.

## Abbreviations

3DCRT: three-dimensional conformal radiotherapy
ANC: absolute neutrophil count
AUC: area under the curve
BM: bone marrow
BMS: bone marrow paring
CCRT: concurrent chemoradiotherapy
CI: confidence interval
CI: confidence interval
C-index: the concordance index
CT: computed tomography
CTCTE: Common Terminology Criteria for Adverse Events
CTV: clinical target volume
D2%, D50% and D99%: the dose of 2%, 50% and 99% of the volume of the target
DCA: Decision Curve Analysis
Dmax: maximize dose
Dmean: mean dose
Dmedian: median dose
Dmin: minimum dose
DSBs: double strand DNA breaks
DVHs: the dose volume histograms
EBRT: external beam radiotherapy
EQD2: equivalent dose in 2 Gy fractions
18FDG-PET: 18 F-fluorodeoxyglucose positron emission tomography
FIGO: International Federation of Gynecology and Obstetrics
GTVnd: gross tumor volume node
HDR: high-dose-rate
HGB: hemoglobin
HI: homogeneity index
HR-CTV: high risk clinical target volume
HT: hematologic toxicity
ICBT: intracavitary brachytherapy
ICRU: International commission on radiation units and measurements
ICRU: International commission on radiation units and measurements
IGBT: image-guided brachytherapy
IMRT: intensity modulated radiotherapy
IG-IMRT: image guided IMRT
IOR: interquartile range
IR: ionizing radiation
KPS: Karnofsky performance status
LACC: locoregionally advanced cervical cancer
LSS: lumbosacral spine
LYM: lymphocyte
NO: nitric oxide
NOXs: nicotinamide adenine dinucleotide phosphate oxidases
ns: no significance
OARs: organs at risk
OR: odds ratio
PB: pelvic bone
PGTVnd: plan gross tumor volume node
PIF: pelvic insufficiency fracture
PLT: platelet
PTV: plan target volume
RCT: randomized controlled trial
ROC: receiver operating characteristic
ROS: reactive oxygen species
RTOG: Radiation Therapy Oncology Group
s.d: standard deviation
SIB: simultaneous integrated boost
SPECT: single photon emission computed tomography
SUV: standardized uptake value
TPS: treatment planning system
VPTV: the target volume
VPTV: ref, the volume of reference isoline wrapped around the target
Vref: the volume of reference isoline wrapped
Vx: volume receiving x Gy
WBC: white blood cell
