# Supplementary material for: Effects of bone marrow sparing radiotherapy on acute hematologic toxicity for patients with locoregionally advanced cervical cancer: a prospective phase II randomized controlled study
Source: Radiat Oncol. 2024 Apr 9;19:46. doi: 10.1186/s13014-024-02432-7 (PMC11005132; doi:10.1186/s13014-024-02432-7)
Supplement: Supplementary file 2 — Supplementary Material 2 [file 13014_2024_2432_MOESM2_ESM.docx]

**Table 1s** CTCAE hematologic toxicity grading system

|  | Grade 1 | Grade 2 | Grade 3 | Grade 4 |
| --- | --- | --- | --- | --- |
| Leukopenia (WBC, *10^9/L) | 3.0 to < LLN | 2.0 to <3.0 | 1.0 to <2.0 | < 1.0 |
| Neutropenia (ANC,*10^9/L) | 1.5 to < LLN | 1.0 to <1.5 | 0.5 to <1.0 | <0.5 or sepsis |
| Anemia (HGB, g/dL) | 95 to < LLN | 75 to <95 | 50 to <75 | <50 |
| Thrombocytopenia (PLT, *10^9/L) | 75 to < LLN | 50 to <75 | 25 to <50 | <25 or spontaneous bleeding |
| Lymphopenia (LYM *10^9/L) | 0.8 to < LLN | 0.2 to <0.8 | 0.2 to <0.5 | <0.2 |

Abbreviation: CTCTE, the Common Terminology Criteria for Adverse Events; WBC, White blood cell; ANC, absolute neutrophil cell; HGB, hemoglobin; LLN, lower limits of normal; PLT, platelet; LYM, lymphocyte.

**Table 2s** Grade ≥2 and grade ≥3 acute HT(with and without lymphopenia), leukopenia, neutropenia, anemia, thrombocytopenia and lymphopenia for patients receiving 45Gy/25f EBRT dose

|  | Grade ≥2 | | | Grade ≥3 | | |
| --- | --- | --- | --- | --- | --- | --- |
|  | BMS (n = 42) | Control (n = 45) | *P* | BMS (n = 42) | Control (n = 45) | *P* |
| HT(without lymphopenia) | 30(71.40%) | 38(84.40%) | 0.142 | 3(7.10%) | 27(60.00%) | *P* <0.001* |
| Leukopenia | 28(66.70%) | 35(77.80%) | 0.247 | 3(7.10%) | 21(46.70%) | *P* <0.001* |
| Neutropenia | 15(35.70%) | 23(51.10%) | 0.148 | 3(7.10%) | 16(35.60%) | 0.003* |
| Anemia | 4(9.50%) | 9(20.00%) | 0.285 | 1(2.40%) | 2(4.40%) | 1.000 |
| Thrombocytopenia | 6(14.30%) | 13(28.90%) | 0.099 | 1(2.40%) | 4(8.90%) | 0.362 |
| Lymphopenia | 42(100.00%) | 44(97.80%) | 1.000 | 39(92.90%) | 36(80.00%) | 0.082 |
| HT(with lymphopenia) | 42(100.00%) | 44(97.80%) | 1.000 | 39(92.90%) | 41(91.10%) | 1.000 |

Abbreviation: HT, hematologic toxicity; BMS, bone marrow sparing. * *P* < 0.05 was considered significant.

**Table 3s** Grade ≥2 and grade ≥3 acute HT(with and without lymphopenia), leukopenia, neutropenia, anemia, thrombocytopenia and lymphopenia for patients receiving 50Gy/25f EBRT dose

|  | Grade ≥2 | | | Grade ≥3 | | |
| --- | --- | --- | --- | --- | --- | --- |
|  | BMS (n = 53) | Control (n = 51) | *P* | BMS (n = 53) | Control (n = 51) | *P* |
| HT(without lymphopenia) | 41(77.40%) | 47(92.20%) | 0.069 | 9(17.00%) | 35(68.60%) | *P* <0.001* |
| Leukopenia | 37(69.80%) | 44(86.30%) | 0.043* | 6(11.30%) | 31(60.80%) | *P* <0.001* |
| Neutropenia | 24(45.30%) | 39(76.50%) | 0.001* | 6(11.30%) | 25(49.00%) | *P* <0.001* |
| Anemia | 13(24.50%) | 20(39.20%) | 0.108 | 3(5.70%) | 5(9.80%) | 0.671 |
| Thrombocytopenia | 14(26.40%) | 18(35.30%) | 0.327 | 3(5.70%) | 3(5.90%) | 1.000 |
| Lymphopenia | 51(96.20%) | 51(100.00%) | 0.495 | 46(86.80%) | 47(92.20%) | 0.568 |
| HT(with lymphopenia) | 53(100.00%) | 51(100.00%) | ns | 47(88.70%) | 50(98.00%) | 0.130 |

Abbreviation: HT, hematologic toxicity; BMS, bone marrow sparing. * *P* < 0.05 was considered significant.

**Table 4s** Grade ≥2 and grade ≥3 acute HT(with and without lymphopenia), leukopenia, neutropenia, anemia, thrombocytopenia and lymphopenia for patients receiving 50.4Gy/25f EBRT dose

|  | Grade ≥2 | | | Grade ≥3 | | |
| --- | --- | --- | --- | --- | --- | --- |
|  | BMS (n = 26) | Control (n = 25) | *P* | BMS (n = 26) | Control (n = 25) | *P* |
| HT(without lymphopenia) | 17(65.40%) | 25(100.00%) | 0.002* | 8(30.80%) | 17(68.00%) | 0.008* |
| Leukopenia | 15(57.70%) | 24(96.00%) | 0.004* | 7(26.90%) | 14(56.00%) | 0.035* |
| Neutropenia | 6(23.10%) | 18(72.00%) | *P* <0.001* | 4(15.40%) | 12(48.00%) | 0.027* |
| Anemia | 4(15.40%) | 8(32.00%) | 0.285 | 1(3.80%) | 4(16.00%) | 0.323 |
| Thrombocytopenia | 5(19.20%) | 9(36.00%) | 0.180 | 1(3.80%) | 1(4.00%) | 1.000 |
| Lymphopenia | 26(100.00%) | 25(100.00%) | ns | 24(92.30%) | 24(96.0%) | 1.000 |
| HT(with lymphopenia) | 26(100.00%) | 25(100.00%) | ns | 24(92.30%) | 25(100.00%) | 0.490 |

Abbreviation: HT, hematologic toxicity; BMS, bone marrow sparing. * *P* < 0.05 was considered significant.

**Table 5s** Descriptive statistics of dosimetric parameters for PTV and OARs

| Parameters | BMS (n = 121) | Control (n = 121) | *P* |
| --- | --- | --- | --- |
| PTV-V(100%) (mean, s.d) | 96.27(1.07) | 96.75(1.29) | 0.002* |
| CI (mean, s.d) | 0.90(0.04) | 0.89(0.04) | 0.134 |
| HI (mean, s.d) | 0.14(0.08) | 0.15(0.07) | 0.364 |
| Small intestine and colon | |  |  |
| V40 (%) (mean, s.d) | 17.24(5.39) | 17.83(5.84) | 0.416 |
| V50 (%) (mean, s.d) | 4.85(2.98) | 4.82(2.95) | 0.934 |
| Rectum-V50 (%) (mean, s.d) | 16.88(12.21) | 22.88(10.32) | P <0.001* |
| Bladder-V50 (%) (mean, s.d) | 23.92(13.42) | 28.44(10.65) | P <0.001* |
| Left femoral head | |  |  |
| V30 (%) (mean, s.d) | 12.81(11.88%) | 21.21(12.85) | P <0.001* |
| V50 (%) (mean, s.d) | 0 | 0 | ns |
| Right femoral head | |  |  |
| V30 (%) (mean, s.d) | 12.07(11.40%) | 20.63(13.42%) | P <0.001* |
| V50 (%) (mean, s.d) | 0 | 0 | ns |
| Left kidney-Dmean (Gy) (mean, s.d) | 10.51(1.53) | 11.02(2.00) | 0.452 |
| Right kidney-Dmean (Gy) (mean, s.d) | 10.46(1.78) | 11.14(1.87) | 0.32 |
| Liver-D30% (Gy) (mean, s.d) | 14.96(1.05) | 14.93(1.94) | 0.727 |
| Spinal cord-V40(%) (mean, s.d) | 0 | 0 | ns |

Abbreviation: PTV, Plan target volume; OARs, organs at risk; BMS, bone marrow sparing; CI, conformity index; HI, homogeneity index; Vx, volume receiving x Gy; Dmean, mean dose; Dx%, dose at x% volume; ns, no significance. * *P* < 0.05 was considered significant.


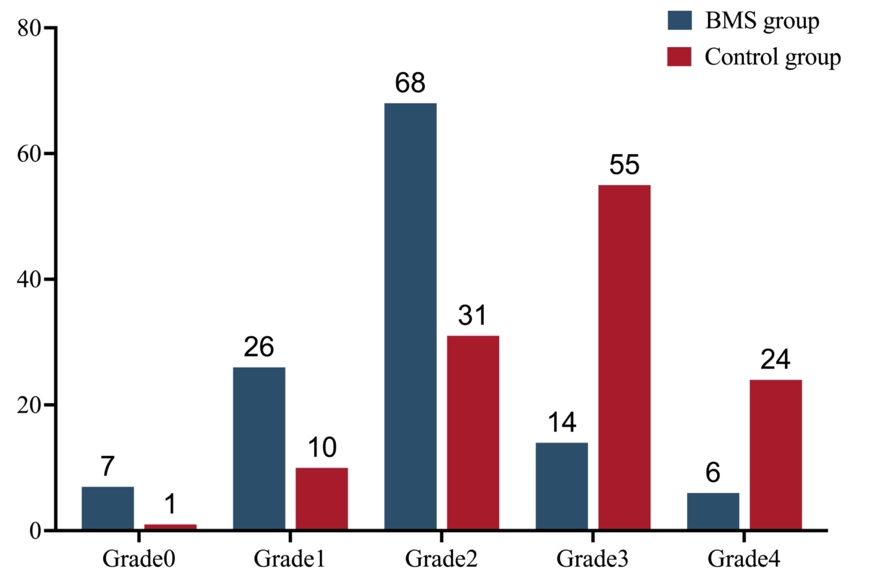


**Fig. 1s** Distribution of 0-4 grade acute HT excluding lymphopenia in the BMS group and control group.

Abbreviation: HT, hematologic toxicity; BMS, bone marrow sparing

A
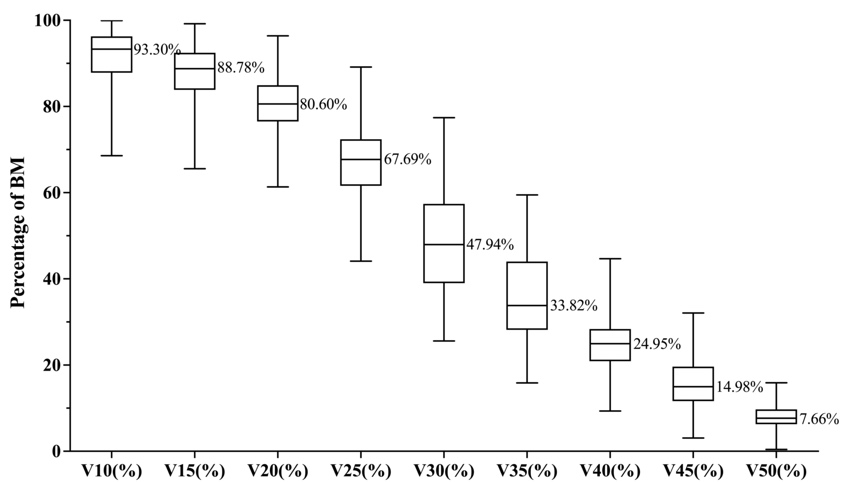


B
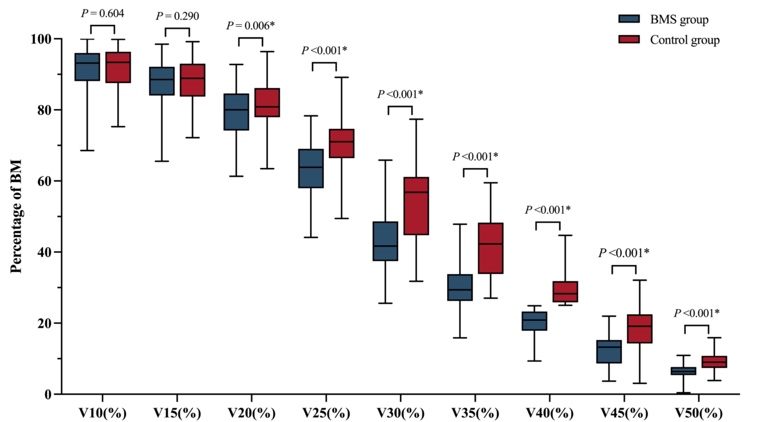


**Fig. 2s** V10(%) to V50(%) of BM for all patients (A) and comparison between the BMS group and the control group.

Abbreviation: Vx,volume receiving x Gy; Dmin, minimum dose; BM, bone marrow; BMS, bone marrow sparing. * *P* < 0.05 was considered significant.
